# An investigation of zinc isotope fractionation in cacao (*Theobroma cacao* L.) and comparison of zinc and cadmium isotope compositions in hydroponic plant systems under high cadmium stress

**DOI:** 10.1038/s41598-023-30899-z

**Published:** 2023-03-22

**Authors:** Elnaz Barati, Rebekah E. T. Moore, Ihsan Ullah, Katharina Kreissig, Barry J. Coles, Jim M. Dunwell, Mark Rehkämper

**Affiliations:** 1 Department of Earth Science and Engineering, Imperial College London, London, UK; 2 School of Agriculture, Policy and Development, University of Reading, Reading, UK

**Keywords:** Plant sciences, Plant physiology, Plant stress responses

## Abstract

This study aims to establish whether zinc (Zn) and cadmium (Cd) share similar physiological mechanisms for uptake and translocation in cacao plants (*Theobroma cacao* L.). Multiple-collector ICP-MS was used to determine the Zn stable isotope compositions in the roots, stems and leaves of 19 diverse cacao genotypes grown in hydroponics with 20 µmol L^−1^ CdCl_2_. Additional plants of one genotype were grown in hydroponic solutions containing lower Cd concentrations (0 and 5 µmol L^−1^ added CdCl_2_). Regardless of the Cd concentration used in the exposures, the Zn stable isotope compositions show the same systematic patterns in plant organs, with δ^66^Zn_root_ > δ^66^Zn_stem_ > δ^66^Zn_leaf_ (δ^66^Zn denotes relative differences in ^66^Zn/^64^Zn ratios in parts per thousand). The mean Zn stable isotope fractionation between the plants and the hydroponic solutions was ε^66^Zn_uptake_ = –1.15 ± 0.36‰ (2SD), indicating preferential uptake of isotopically light Zn by plants from the hydroponic solution. The mean  stable isotope fractionation factor associated with translocation of Zn from roots to shoots, ε^66^Zn_seq-mob_ =  + 0.52 ± 0.36‰ (2SD), shows that isotopically heavy Zn is preferentially sequestered in the cacao roots, whilst isotopically light Zn is mobilised to the leaves. A comparison with the Cd stable isotope compositions of the same plants shows that both isotopically light Zn and Cd are preferentially taken up by cacao plants. In contrast to Zn, however, the cacao roots retain isotopically light Cd and transfer isotopically heavy Cd to the leaves.

## Introduction

Zinc (Zn) is one of the most abundant essential micronutrients in organisms, and the only trace metal required for all the six major enzyme classes^1, 2^. As a co-factor for over 300 enzymes^3^, Zn is involved in enzyme activity regulation, transcriptional and translational control functions, protein-binding, and signal transduction^4–6^. Due to it playing a vital role in plant physiology and plant cell growth and development, Zn needs to be maintained at homeostasis in plant cells^4, 6^. This process involves Zn concentrations in plant cells being maintained within a narrow and specific range to avoid deficiency or toxicity effects during changing conditions^4, 6, 7^.

Understanding the uptake and translocation of Zn can provide invaluable insights into the physiological mechanisms that are vital for plant growth and development^4–6, 8^. Previous studies that examined metal hyperaccumulators and metal tolerant plants^9–17^, fruit, vegetable and cereal crops^18–21^, and other plant species^22–24^ have shown that stable isotope analyses can provide insight into the mechanisms employed by plants to regulate the uptake of Zn and its translocation from the roots to the aerial plant organs^25^. Differences in Zn stable isotope compositions between plant species and internal organs occur due to variations in physiological mechanisms that are responsible for the uptake of a metal from its source (e.g., hydroponic solution or soil) and its distribution and accumulation in different plant organs (e.g., roots, stems, leaves and seeds/fruits)^25, 26^. Physiological mechanisms such as root uptake, short- and long-distance transport, metal storage and compartmentalization, and metabolic functionalization are controlled by mechanisms such as sorption, ligand exchange, redox changes, precipitation, and diffusion^25^. As such, through stable isotope analyses, inferences can be made about mechanisms, including the primary binding form in which Zn is retained in a particular plant organ^25–27^. Most previous studies have shown, for example, that isotopically heavy Zn is retained in plant roots, whilst isotopically light Zn is preferentially translocated to and stored in aerial plant organs^25^. In addition, stable isotope analyses of different metals in a plant sample can provide insights into interactions among metals with similar physical and chemical properties^26^.

As a trace heavy metals of group 12 of the periodic table, Zn and cadmium (Cd) share similar chemical and physical properties. Due to this similarity, plant species may use similar mechanisms for regulating the uptake and translocation of Zn and Cd^4, 7, 31–33^. However, the underlying mechanisms responsible for regulating Zn and Cd in different plant species are not fully understood and it is hence unclear if different plant species share similar uptake and translocation mechanisms^4, 28–34^. For example, previous studies have shown that the application of Zn to hydroponic solutions or soils can either reduce or increase the uptake and translocation of Cd, depending on the plant species^28–34^. In comparison to the essential micronutrient Zn, Cd is a known enzyme inhibitor in plants, and in plant cells it can cause mitochondrial damage, cell proliferation and cell division inhibition, lipid peroxidation, DNA and RNA degradation, ion leakage and cell membrane damage^4, 33^. Damage to cell organelles in plants is related to exposure to high Cd concentrations (e.g., 50–100 mg kg^−1^) in nutrient growth media (e.g., hydroponic solutions and soils), but the specific Cd concentration threshold above which damage occurs is highly dependent on the plant species^35^. In humans, progressive accumulation of Cd in the body through long-term exposure to higher Cd levels in air, water, soils, and food can lead to adverse health complications^36, 37^. These well-documented complications include permanent damage to the urinary and skeletal systems and various cancers^36^.

Irrespective of their age, cacao plants are considered as Cd “accumulators” because they have the capacity to accumulate high amounts of Cd without impacting their growth and development^38–42^. Barraza et al.^39^, for instance, found that banana, pineapple, lemon and soursop grown alongside cacao in Ecuador had fruit with Cd concentrations of up to 0.022 µg g^−1^, whilst the Cd levels in cacao beans were much higher at between 0.1 and 1.6 µg g^−1^. Elsewhere in South and Central America, Cd concentrations of between 1 and 10 μg g^−1^ are common for cacao beans^38–43^. These high Cd concentrations make it increasingly challenging for cacao-based industries to meet the strict Cd concentration limits set by the EU in 2019 (maximum of 0.1–0.8 µg g^−1^ Cd) for final cacao-based products^37, 44^.

Cadmium stable isotope analyses have helped to constrain and understand the underlying mechanisms responsible for the uptake of Cd and its translocation to cacao beans^45, 46^. In particular, previous Cd stable isotope studies suggest that cacao plants preferentially remove isotopically light Cd from hydroponic solutions using membrane transporter proteins such as natural resistance-associated macrophage proteins (NRAMPs). In addition, it was shown that cacao plants retain isotopically light Cd in the roots, whilst translocating isotopically heavy Cd to aerial parts, possibly due to regulation by proteins such as heavy metal ATPases (HMAs)^46^. To date, however, no studies have directly determined the Zn stable isotope compositions of cacao plants. Such stable isotope data will not only provide insight into how Zn is regulated and translocated in cacao but can also reveal whether the physiological mechanisms of Zn regulation are impacted by Cd. In this study, juvenile cacao plants were used to investigate whether the overarching mechanisms responsible for Zn uptake and translocation are similar to those of Cd and whether these mechanisms change under high Cd conditions. This is the first step towards understanding the mechanisms responsible for the uptake of Zn and its translocation to cacao beans in mature cacao plants under field conditions. Subsequently, a better understanding of both metals can be used to inform future agricultural and land management practices that aim to reduce Cd accumulation in cacao, and particularly cacao beans, without disrupting Zn homeostasis. For example, if Cd and Zn share and possibly compete for the same physiological mechanisms responsible for root uptake, Zn applied through fertilizers could be used to reduce the overall uptake of Cd by the cacao plant and its subsequent translocation to cacao beans^47^. In contrast, if the metals do not share similar mechanisms, then this could also be used to design genetically distinct cultivars that allow sufficient Zn to reach the important organs but exclude Cd or sequester the metal in organs other than the beans^47, 48^.

In this study, hydroponic plant systems were used to grow 19 genetically distinct varieties of cacao. Hydroponic plant studies uniquely allow the testing of plant responses to different nutrient availabilities and exposure to toxic metals, through controlling the concentrations of the metal of interest, in this case, Zn and Cd. In addition, the systems also reduce confounding factors observed in soil studies from soil microenvironments^49, 50^. This controlled set-up enables direct analysis of how Cd concentrations in the hydroponic solution influence the uptake and translocation of Zn in cacao plants. Most importantly, hydroponic plant systems allow for root and shoot tissues to be easily harvested and separated for analyses of the entire plant system^49^.

This study is focused on examining the uptake and translocation of Zn and its stable isotopes in Cd-stressed juvenile cacao plants grown under controlled hydroponic conditions. Stable isotope analyses were conducted on the leaves, stems and roots of 19 diverse cacao genotypes; the same plants previously analysed by Moore et al.^46^ for Cd stable isotope compositions. The aims of the current investigation were to (1) examine the Zn  stable isotope fractionation among the plant organs across the 19 genotypes to investigate overarching mechanisms for Zn uptake and translocation; (2) compare Zn and Cd stable isotope fractionation to identify shared or distinct physiological mechanisms for the uptake and translocation of these metals across the 19 genotypes; and (3) examine whether the availability of Cd influences the uptake of Zn by cacao plants and its translocation to aerial plants organs.

## Methods

### Samples

#### Cultivation of cacao plants

Juvenile cacao plants representing 19 different genotypes were obtained from the International Cocoa Quarantine Centre at the University of Reading, UK (Supplementary Table S1). These plants were identical to those analysed in the study of Moore et al.^46^. The planting, growing, and harvesting period of the juvenile cacao plants encompassed a total of 8 weeks. All cacao plants were grown under controlled environmental conditions, with temperatures of 28 °C during the day and 20 °C at night with a 16-h photoperiod at a 60% relative humidity. The cacao seeds (nibs) first had their coats (testa) removed and then were placed in seed compost for germination. After two weeks, as part of the hydroponic system, the seedlings were transferred into food grade polyethylene containers (Clas Ohlson) with a hydroponic solution comprising 3 L of half-strength Hoagland solution with a pH of 5.2 (Supplementary Table S2). A sufficient amount (0.38 μmol L^−1^) of zinc sulfate (ZnSO_4_·7H_2_O) was added as part of the half-strength Hoagland solution. In each container, there were four cacao seedlings of the same genotype. The hydroponic solution was aerated for 15 min every 2 h and renewed every 7 days.

After 28 days, the hydroponic solutions were continued to be renewed for a further 14 days, but with the addition of 20 µmol L^−1^ CdCl_2_ (Sigma-Aldrich, 99.99% trace metals basis). To assess to what extent different Cd concentrations influenced plant growth and the uptake and translocation of Zn, additional seedlings of one genotype (NA 702) were grown under the same conditions as the other seedlings, but only 0 and 5 µmol L^−1^ CdCl_2_ were added to the hydroponic solutions for the final 14 days. The NA 702 genotype, part of Nanay family, was selected for the additional Cd conditions as it is one of the most widely used and traditionally grown cacao genotypes in South and Central America^51^.

After 14 days of Cd stress, the plants were harvested and separated into leaves, stems and roots. To remove any residual Zn, Cd and other metals that were apoplastically bound to the roots, the roots were bathed in a solution of 20 mmol L^−1^ Na_2_EDTA for 15 min and then rinsed with deionised water. The leaves and stems were washed with deionised water. After cleaning, the different plant organs were oven-dried at 70 °C and ground using a plastic grinder. Between each sample grinding, the grinder was cleaned with 18.2 MΩ cm^−1^ grade (Milli-Q) H_2_O and ethanol.

#### Plastic leaching experiment

Part of the experimental conditions by which the cacao plants were grown was replicated to understand the contribution of Zn from plastic leaching to the total concentration of Zn in the hydroponic solutions. Two additional hydroponic containers were filled with 3 L solutions of 0.25 µg L^−1^ Zn with a pH of about 6. These solutions were prepared using the same ZnSO_4_ as used in the plant experiments and Milli-Q H_2_O. The plastic tubing, foam stoppers and vials used to hold and grow the cacao seedlings, were also placed in the containers. A lower Zn concentration (0.25 µg L^−1^) than present in the hydroponic solutions was used here, so that any Zn addition, particularly from leaching of plastics, would be easy to detect and quantify. To replicate the hydroponic systems, the solutions were renewed each week for 6 weeks and before each renewal, a 15 mL aliquot was taken from each solution and stored in a Savillex Teflon vial. The aliquots from each week were combined, dried down on a hotplate and then treated with 6 mol L^−1^ HCl to convert Zn into the chloride form.

### Sample preparation

Sample preparation was performed in the clean room facilities of the MAGIC Laboratories, Imperial College London. The mineral acids used in this study, 15.4 mol L^−1^ HNO_3_, 5.8 mol L^−1^ HCl and 28 mol L^−1^ HF, were prepared by sub-boiling distillation in Teflon or quartz glass stills from AnalaR grade stock acids. All acid dilutions used Milli-Q H_2_O from a Millipore purification system. Samples were stored in acid-cleaned Savillex Teflon vials.

#### Digestion

Between 50 and 550 mg of the leaf, stem and root samples alongside the reference material NIST spinach leaf SRM 1570a, were digested in 100 mL PTFE vessels with 7 mL of 15.4 mol L^−1^ HNO_3_ and 3 mL of 30–32% Optima grade H_2_O_2_ (Romil) using a Milestone Ethos EZ microwave system. The treated samples of SRM 1570a were matched to the sample weights of the different plant organs that were digested for analysis. The digestion protocol consisted of heating at 90 °C for 15 min and then at 180 °C for 30 min. After completion, the samples were transferred to 15 mL Savillex Teflon vials and dried on a hotplate. Then, 700 µL of 15.4 mol L^−1^ HNO_3_, 300 µL of H_2_O_2_ and 20–50 µL of 28 mol L^−1^ HF were added and the digestion continued at 120 °C on a hotplate for approximately 48 h, until all residual organic materials and silica were completely dissolved.

Digestions were carried out for biological and analytical replicates of plants to determine their homogeneity and biological variability. These constituted single leaf, stem, and root samples of three separate plants of genotypes GU 207/H and CC 41, and duplicate analyses of leaf, stem, and root samples from a single plant of Matina 1–7.

#### Zinc separation chemistry

Following digestion, appropriate aliquots of the sample solutions were further treated for subsequent Zn stable isotope measurements. For each set of eight sample digestions, a procedural blank was prepared. For the stable isotope measurements, sample aliquots with about 250 ng Zn were mixed with an appropriate volume of a ^64^Zn–^67^Zn double spike (DS) solution to obtain a ratio of DS-derived to natural Zn (S/N) of about 1–1.2, and then equilibrated overnight at 120 °C^52, 53^. To optimise the S/N ratio, the Zn concentrations of the samples were determined by a multiple-collector inductively-coupled mass spectrometer (MC-ICP-MS) prior to this step using a ~ 10% aliquot of the sample solutions. Once equilibrated with the DS, anion exchange chromatography was utilized to prepare purified Zn fractions from the samples^52^. This procedure consisted of filling shrink-fit Teflon columns with 3 mL acid reservoirs with 250 μL of the anion-exchange resin (AG MP-1 M, 100–200 mesh). After cleaning with 8 mL of 0.1 mol L^−1^ HNO_3_ and 0.5 mL of MQ H_2_O, the resin was converted to Cl^−^ form using 3.5 mL of 6 mol L^−1^ HCl. Following this, 2 mL of 1 mol L^−1^ HCl was added. The samples, as well as a procedural blank and a column blank, were then loaded onto the resin columns as solutions in 1 mL of 1 mol L^−1^ HCl. The sample matrix was eluted with 8 mL of 1 mol L^−1^ HCl, whilst the Zn fractions were then stripped from the resin by adding 6 mL of 0.01 mol L^−1^ HCl. Once the Zn fractions were isolated, they were dried, dissolved in a few drops of 15.4 mol L^−1^ HNO_3_ and re-dried, twice, to remove Cl^−^. Finally, the samples were dissolved in 0.1 mol L^−1^ HNO_3_ to make solutions of 100 ng mL^−1^ Zn for analyses via MC–ICP–MS.

### Zinc concentration and stable isotope measurements

The Zn stable isotope compositions of the sample were determined in the MAGIC Laboratories using a Nu Instruments Nu Plasma HR MC-ICP-MS. Sample introduction to the MC–ICP–MS was performed with a Cetac autosampler and Cetac Aridus II desolvation system fitted with a glass nebuliser that had nominal uptake rates of 100 µL min^−1^. A Faraday collector configuration that employed the L5, L3, Ax, H2, H3 and H4 cups was used for the analyses, with all collectors fitted with 10^11^ Ω resistors. Faraday collectors L3, Ax, H2 and H4 were employed to measure the ion beams of ^64^Zn, ^66^Zn, ^67^Zn and ^68^Zn, while L5 and H3 were used to monitor ^62^Ni and ^135^Ba^2+^ to allow for spectral interference corrections of isobaric Ni^+^ and Ba^2+^ ions on the different Zn isotopes. Each sample measurement entailed three blocks of twenty 5 s integration cycles, with electronic baselines measured for 15 s prior to each block, during which the ion beam was deflected in the electrostatic analyser^52^. For sample measurements, the instrumental sensitivity for Zn was typically between 120 and 150 V/(µg/mL).

As MC-ICP-MS is prone to instrumental drift, the sample runs were bracketed by analyses of the AA-ETH Zn isotope standard^54^. The AA-ETH Zn solutions had S/N values and Zn concentrations that matched the values of the samples to within 10%. The analytical precision of the Zn stable isotope data for samples was determined from the precision of the bracketing analyses of the AA-ETH Zn isotope standard that were conducted in the same measurement session. Such analyses yielded analytical precisions that were typically between ± 0.02 and ± 0.09‰ (Table 1). The secondary London Zn isotope standard and spinach leaf reference material NIST SRM 1570a were also analyzed alongside samples to further monitor data quality.

**Table 1:** Zinc mass fractions (*f*), concentrations (mg kg^−1^) and stable isotope compositions (δ^66^Zn) of leaves, stems and roots for 19 cacao genotypes grown with 20 µmol L^−1^ CdCl_2_.

Cacao clones (genotype)	Leaf				Stem				Root			
	*f*	[Zn] (mg kg^−1^)	δ^66^Zn (‰)	2SD (‰)	*f*	[Zn] (mg kg^−1^)	δ^66^Zn (‰)	2SD (‰)	*f*	[Zn] (mg kg^−1^)	δ^66^Zn (‰)	2SD (‰)
B 5/7 [POU]	0.69	21	− 0.74	0.05	0.22	15	− 0.50	0.05	0.09	16	0.68	0.07
Catie 1000	0.58	28	− 0.67	0.02	0.29	35	− 0.27	0.08	0.13	15	0.50	0.04
CC 41^a^	0.72	27	− 0.66	0.05	0.20	19	− 0.18	0.07	0.08	14	0.51	0.04
CL 19/10	0.48	14	− 0.76	0.05	0.45	62	0.25	0.07	0.06	16	0.52	0.02
GU 207/H^a^	0.73	32	− 0.43	0.04	0.15	28	− 0.42	0.07	0.12	16	0.40	0.05
GU 236/V	0.70	34	− 0.52	0.06	0.16	28	− 0.13	0.06	0.14	16	0.45	0.08
IMC 27	0.70	43	− 0.95	0.05	0.20	25	− 0.36	0.09	0.10	13	0.41	0.09
LP 1/41 [POU]	0.64	22	− 0.71	0.09	0.28	23	− 0.46	0.08	0.08	17	− 0.28	0.06
Matina 1–7^b^	0.60	20	− 0.73	0.08	0.31	28	− 0.63	0.06	0.09	15	− 0.02	0.08
NA 702	0.71	16	− 0.70	0.08	0.20	16	− 0.28	0.07	0.09	12	0.40	0.06
PNG 340	0.60	16	− 0.79	0.06	0.32	14	− 0.67	0.08	0.08	10	0.11	0.06
Pound 12/A [POU]	0.68	26	− 0.95	0.05	0.21	30	− 0.31	0.07	0.10	17	0.29	0.00
RB 46	0.55	15	− 1.11	0.08	0.28	29	− 0.35	0.08	0.17	18	0.39	0.05
RIM 179	0.71	23	− 0.72	0.06	0.18	23	− 0.50	0.05	0.11	15	− 0.06	0.02
SCA 9	0.69	28	− 0.64	0.09	0.21	22	− 0.34	0.09	0.10	23	0.59	0.02
SPA 9 [COL]	0.65	25	− 0.80	0.07	0.24	23	− 0.35	0.09	0.12	19	0.55	0.02
TARS 31	0.62	30	− 0.72	0.04	0.21	30	− 0.09	0.09	0.17	16	0.54	0.05
TSA 654	0.73	31	− 0.72	0.09	0.19	33	− 0.25	0.01	0.07	14	0.41	0.07
U 70 [PER]	0.79	63	− 0.43	0.09	0.13	31	0.11	0.06	0.08	23	0.67	0.07
Min	0.48	14	− 1.11	0.02	0.13	14	− 0.67	0.01	0.06	10	− 0.28	0.00
Max	0.79	63	− 0.43	0.09	0.45	62	0.25	0.09	0.17	23	0.68	0.09
Mean	0.66	27	− 0.72	–	0.23	27	− 0.30	–	0.10	16	0.37	–
1SD	0.07	11	–	–	0.07	10	–	–	0.03	3	–	–
2SD	–	–	0.32	–	–	–	0.44	–	–	–	0.50	–

^a^Mean values for the leaf, stem, and root aliquots from three separate plants of the CC 41 and GU207/H genotypes (*n* = 3).

^b^Mean value for the duplicate leaf, stem, and root aliquots from a single plant of the Matina 1–7 genotype (*n* = 1). *f * represents the mass fraction of Zn in leaves, stems, and roots relative to the Zn inventory of the total plant. SD represents the standard deviation. 2SD for individual samples represents the analytical precision of the Zn stable isotope data determined from multiple analyses of the Zn isotope standard that bracketed the sample runs.

Following data acquisition, the raw measured isotope ratios were treated offline to correct for both spectral interferences and any  instrumental and laboratory-induced mass bias using an iterative  DS data reduction method^53, 55, 56^. The Zn concentrations of the samples were determined with the isotope dilution technique from the mass bias corrected isotope data.

### Calculations and data analysis

The Zn stable isotope compositions of the samples are reported in δ notation (‰). The δ values were originally determined relative to data obtained in the same measurement session for bracketing runs of the AA-ETH Zn isotope standard:

1 $${\updelta }^{66}\mathrm{Zn}=\left[ \left(\frac{{(}^{66}\mathrm{Zn}{/}^{64}\mathrm{Zn}{)}_{\mathrm{sample}} }{{(}^{66}\mathrm{Zn}{/}^{64}\mathrm{Zn}{)}_{\mathrm{AA}-\mathrm{ETH Zn}}}\right)-1\right]\mathrm{ x }1000$$

In the following, all δ^66^Zn values are reported relative to the JMC-Lyon Zn isotope standard. To this end, the initial results were corrected for a δ^66^Zn offset of + 0.28‰ for AA-ETH Zn relative to JMC-Lyon Zn^54^.

For the comparison of Zn stable isotope compositions, the apparent isotope fractionation or difference between two samples or reservoirs, denoted by A and B, was calculated as:

2 $${\Delta }^{66}{\mathrm{Zn}}_{\mathrm{A}-\mathrm{B}}= {\updelta }^{66}{\mathrm{Zn}}_{\mathrm{A}}- {\updelta }^{66}{\mathrm{Zn}}_{\mathrm{B}}$$

A one-way analysis of variance (ANOVA) was conducted for the Zn stable isotope fractionations that were determined for cacao plants treated with different concentrations of CdCl_2_ (0, 5 and 20 µmol L^−1^). The level of significance for the ANOVA test was set at p < 0.05. Further ANOVA and Kruskal–Wallis tests were conducted for the dry biomass (g), Zn concentrations (mg kg^−1^) and δ^66^Zn values of the cacao organs from the 19 genotypes, with the level of significance also set at p < 0.05. The coefficient of determination (R^2^) was calculated for the dry biomass (g), Zn concentrations (mg kg^−1^) and δ^66^Zn values of the 19 cacao genotypes.

### Ethical approval

All research complied with all relevant institutional, national, and international guidelines and legislation.

## Results

### Quality control

#### Blank contributions and reference materials

The total procedural Zn blank during the study was between 1 and 10 ng (*n* = 41). This corresponds to mean and maximum blank contributions of 0.6% and 1.9%, respectively, to the total Zn mass of the cacao samples. All Zn concentrations were blank corrected to account for exogenous Zn contributions, but no corrections were applied to the δ^66^Zn data as the stable isotope composition of the blank is not well defined. For the full 6-week growing period of the hydroponic experiment, the total contribution of exogenous Zn from plastic leaching to the Zn concentration of the hydroponic solution accounted to 2.0%. The δ^66^Zn value of the solution in the hydroponic set-up (+ 0.47 ± 0.03‰, 2SD*; n* = 2) was, furthermore, identical (within analytical precision) to that of the ZnSO_4_ reagent (+ 0.51 ± 0.06‰, 2SD; *n* = 7). Given these findings, the hydroponic solutions were assigned an isotope composition of δ^66^Zn =  + 0.51 ± 0.06‰, as there were more analyses for the ZnSO_4_ reagent.

The Zn stable isotope and concentration data obtained for the secondary isotope standard and spinach leaf SRM 1570a showed excellent reproducibility and agreed with the published literature values (Supplementary Table S3). In detail, replicate runs of the secondary London Zn isotope standard yielded a mean δ^66^Zn of + 0.10 ± 0.06‰ (2SD; *n* = 30) whilst multiple analyses of SRM 1570a defined a mean result of δ^66^Zn =  + 0.41 ± 0.06‰ (2SD; *n* = 19).

#### Zinc data for biological and analytical replicates of cacao plant samples

Zinc concentrations, [Zn], were determined for samples from three different plants of the CC 41 and GU 207/H genotypes and duplicate samples from a single Matina 1–7 plant. These replicates displayed limited variability with coefficients of variation (CV) of 17%, 12% and 7% for the whole plant Zn concentrations of CC 41, GU 207/H and Matina 1–7, respectively (Tables 1, 2 and Supplementary Table S4). Considering the  stable isotope data, with one exception, all δ^66^Zn values determined for replicate leaf, stem and root samples of the CC 41, GU 207/H and Matina 1–7 plants were identical, within uncertainty, with one another (Tables 1, 2 and Supplementary Table S4). The exception is a single leaf aliquot from the CC 41 genotype, which was isotopically heavier by about 0.35‰ compared to the other two CC 41 leaf aliquots (Supplementary Table S4). These findings are in accord with the results of previous studies that employed plants grown in hydroponic systems under controlled conditions, and which demonstrate that such experiments typically produce plants that yield reproducible results for metal uptake and translocation^11, 12, 18^. These previous and our current results thus indicate that additional replicate analyses for the different cacao genotypes of this study are not needed to arrive at robust conclusions, also considering that  stable isotope measurements are particularly time-consuming and expensive.

**Table 2:** Zinc masses (µg), concentrations (mg kg^−1^) and stable isotope compositions (δ^66^Zn) for total plants, and Zn stable isotope differences (Δ^66^Zn) and fractionation factors (ε^66^Zn).

Cacao clones (genotype)	Total plant					Isotopic differences^d^			Fractionation factors	
	Zn (µg)	[Zn] (mg kg^−1^)	δ^66^Zn (‰)	2SD (‰)	Zn Uptake^c^ (%)	Δ^66^Zn_total-sol_^d^ (‰)	Δ^66^Zn_leaf-total_^d^ (‰)	Δ^66^Zn_shoot-total_^d^ (‰)	ε^66^Zn_uptake_^e^ (‰)	ε^66^Zn_seq-mob_^f^ (‰)
B 5/7 [POU]	17	19	− 0.56	0.04	14.7	− 1.07	− 0.18	− 0.06	− 1.16	0.48
Catie 1000	15	27	− 0.40	0.03	12.8	− 0.91	− 0.27	− 0.06	− 0.98	0.49
CC 41^a^	21	23	− 0.46	0.04	18.7	− 0.98	− 0.19	− 0.04	− 1.09	0.58
CL 19/10	39	22	− 0.22	0.04	34.4	− 0.73	− 0.54	− 0.03	− 0.91	0.74
GU 207/H^a^	18	29	− 0.33	0.03	15.3	− 0.84	− 0.10	− 0.05	− 0.92	0.32
GU 236/V	16	28	− 0.32	0.04	13.9	− 0.83	− 0.20	− 0.06	− 0.89	0.56
IMC 27	19	32	− 0.70	0.04	16.2	− 1.21	− 0.25	− 0.04	− 1.33	0.71
LP 1/41 [POU]	21	22	− 0.61	0.06	18.7	− 1.12	− 0.10	0.02	− 1.24	0.23
Matina 1–7^b^	45	21	− 0.63	0.05	39.4	− 1.15	− 0.09	0.00	− 1.49	0.18
NA 702	27	16	− 0.52	0.06	23.7	− 1.03	− 0.18	− 0.03	− 1.18	0.53
PNG 340	24	14	− 0.68	0.04	21.2	− 1.19	− 0.11	− 0.01	− 1.34	0.22
Pound 12/A [POU]	20	25	− 0.69	0.04	17.3	− 1.20	− 0.26	− 0.03	− 1.32	0.70
RB 46	14	18	− 0.64	0.05	12.5	− 1.15	− 0.47	− 0.07	− 1.23	0.78
RIM 179	42	22	− 0.61	0.04	36.8	− 1.12	− 0.11	0.01	− 1.42	0.32
SCA 9	27	26	− 0.45	0.06	23.7	− 0.96	− 0.19	− 0.06	− 1.10	0.50
SPA 9 [COL]	21	24	− 0.53	0.05	18.2	− 1.04	− 0.27	− 0.06	− 1.16	0.61
TARS 31	26	26	− 0.37	0.03	22.3	− 0.88	− 0.35	− 0.09	− 1.00	0.73
TSA 654	26	29	− 0.55	0.07	22.8	− 1.06	− 0.17	− 0.03	− 1.21	0.56
U 70 [PER]	20	50	− 0.27	0.07	17.2	− 0.78	− 0.16	− 0.05	− 0.86	0.67
Min	14	14	− 0.70	–	12.5	− 1.21	− 0.54	− 0.09	− 1.49	0.18
Max	45	50	− 0.22	–	39.4	− 0.73	− 0.09	0.02	− 0.86	0.78
Mean	24	25	− 0.50	–	21.0	− 1.01	− 0.22	− 0.04	− 1.15	0.52
1SD	8.8	7.4	–	–	7.7	–	–	–	–	–
2SD	–	–	0.29	–	–	0.15	0.24	0.06	0.36	0.36
NA 702 (0 µmol L^−1^ CdCl_2_)^g^	30	16	− 0.38	0.04	26.0	− 0.89	− 0.12	− 0.04	− 1.04	0.25
NA 702 (5 µmol L^−1^ CdCl_2_)^g^	26	15	− 0.39	0.05	22.8	− 0.90	− 0.03	− 0.02	− 1.03	0.09
NA 702 (20 µmol L^−1^ CdCl_2_)^g^	27	16	− 0.52	0.06	23.7	− 1.03	− 0.18	− 0.03	− 1.18	0.53
Mean	28	16	− 0.43	–	24.5	− 0.92	− 0.10	− 0.03	− 1.06	0.29
1SD	2	1	–	–	3.12	–	–	–	–	–
2SD	–	–	0.12	–	–	0.18	0.14	0.04	0.20	0.37

^a^Mean values for the leaf, stem, and root aliquots of three separate CC 41 and GU 207/H plants (*n* = 3).

^b^Mean value for the duplicate leaf, stem, and root aliquots of a single Matina 1–7 plant (*n* = 1).

^c^Fraction of Zn (in %) taken up from the hydroponic solutions by the 4 cacao plants of the same genotype cultured in the same hydroponic container.

^d^Isotopic differences between total plants and the ZnSO_4_ added to the hydroponic solutions (Δ^66^Zn_total-sol_), between leaves and total plants (Δ^66^Zn_leaf-total_), and between shoots and total plants (Δ^66^Zn_shoot-total_). SD represents the standard deviation. 2SD for individual samples represents the analytical precision of the Zn stable isotope data determined from multiple analyses of the Zn isotope standard that bracketed the sample runs.

^e^ε^66^Zn_uptake_ denotes stable isotope fractionation factor for the uptake of Zn by the cacao plants from the hydroponic solutions.

^f^ε^66^Zn_seq-mob_ represents the Zn stable isotope fractionation between the sequestered and mobile Zn in cacao plant.

^g^The NA 702 genotypes treated with different CdCl_2_ concentrations had different samples sizes: 0 µmol L^−1^ CdCl_2_, n = 3; 5 µmol L^−1^ CdCl_2_, n = 2; 20 µmol L^−1^ Cl_2_, n = 1.

### Zinc data for the 19 cacao genotypes

**Figure 1:**
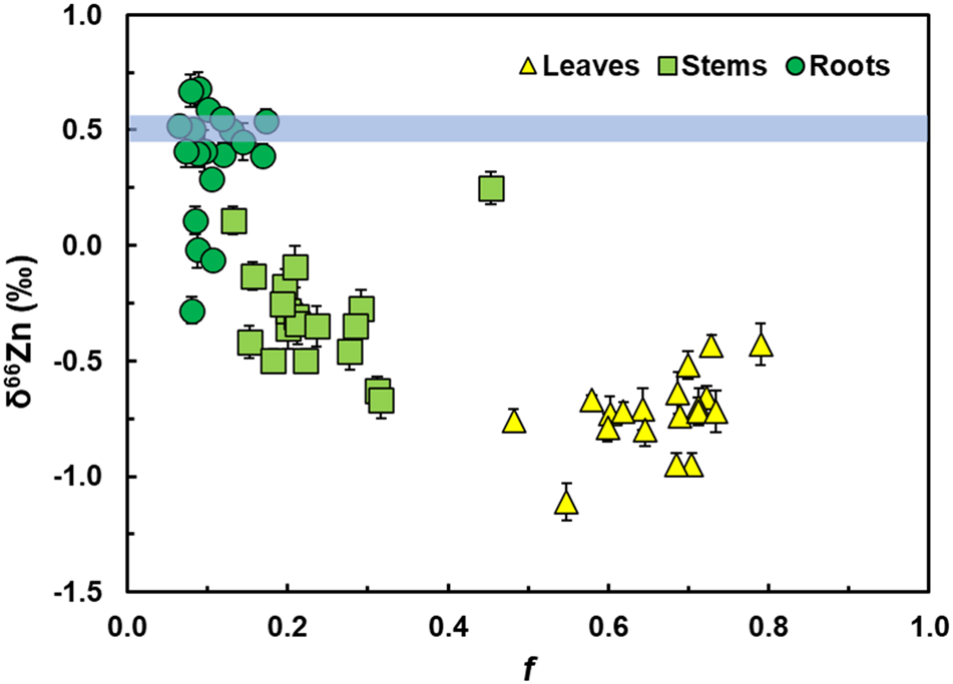
Zinc stable isotope compositions (δ^66^Zn) of leaves, stems and roots versus mass fractions (*f*) of Zn in leaves, stems and roots for the 19 cacao genotypes treated with 20 µmol L^−1^ CdCl_2_. The error bars denote the 2SD precision determined for multiple analyses of the Zn isotope standard that bracketed the sample runs. The blue line denotes the δ^66^Zn value of + 0.51 ± 0.06‰ for the ZnSO_4_ that was added to the hydroponic solutions.

The dry plant masses are summarized in Supplementary Table S5. The cacao seedlings grew to a total dry mass (root, stem, leaves) of between 0.40 g and 2.16 g, with roots, stems and leaves accounting for 14–17%, 21–26%, and 62–64%, respectively. For all 19 cacao genotypes, the Zn contents of the plant organs (Zn_leaf_, Zn_stem,_ and Zn_root_) and the mass fractions of Zn in each organ relative to the total plant Zn inventory (*f*_leaf_, *f*_stem_ and *f*_root_) followed the pattern leaf > stem > root (Tables 1, 2, Fig. 1 and Supplementary Fig. S1). The mean Zn mass fractions were *f*_leaf_ = 66.3%, *f*_stem_ = 23.3%, and *f*_root_ = 10.4% (Table 1), and the total Zn inventory of the plants, Zn_total plant_, was positively correlated with total biomass (R^2^ = 0.78, p < 0.05).

The [Zn]_total plant_ data ranged between 14 and 50 mg kg^−1^ (Table 2) and these show a strong positive correlation with [Zn]_leaf_ (R^2^ = 0.93, p < 0.05; Supplementary Fig. S2), which indicates that [Zn]_total plant_ is controlled by [Zn]_leaf_. The Zn concentrations for the cacao plant organs of the 19 genotypes were variable. For 17 genotypes, [Zn]_leaf_ exceeded [Zn]_root_, whilst only for 9 genotypes [Zn]_leaf_ exceeded [Zn]_stem_. Furthermore, for all but two of the 19 genotypes, [Zn]_stem_ exceeded [Zn]_root_. The Zn concentrations ranged from 14 to 63 mg kg^−1^ for [Zn]_leaf_, 14 to 62 mg kg^−1^ for [Zn]_stem_, and 10 to 23 mg kg^−1^ for [Zn]_root_. The [Zn]_leaf_ values were in accord with literature data for mature leaves from cacao plants grown in the field, and this indicates that the cacao plants of our study received sufficient Zn from the hydroponic solutions (Supplementary Table S6). The cacao genotype U70 [PER] had the highest [Zn]_leaf_ and [Zn]_root_ (tied with SCA 9 for highest [Zn]_root_), whereas genotype CL19/10 had the highest [Zn]_stem_. The cacao genotypes CL 19/10 had the lowest [Zn]_leaf_ and PNG 340 had the lowest [Zn]_stem_ and [Zn]_root_. Overall, [Zn]_root_ had the lowest variability, with a CV of 20%, whereas [Zn]_leaf_ and [Zn]_stem_ were more variable and had similar concentration ranges, with CVs of 42% and 38%, respectively (Table 1 and Supplementary Fig. S1). No strong relationships (R^2^ > 0.5, p < 0.05) were observed between the Zn concentrations of the different organs or with the dry biomass of the different organs.

Over the course of the full 6-week growing period of the hydroponic experiment, the four cacao plants in each hydroponic setup depleted the ZnSO_4_ added to the hydroponic solution by, on average, 21% (Table 2). This depletion factor was determined from the total Zn inventory of the plants in each hydroponic setup relative to the total amount of Zn that was available to the plants via the provided hydroponic solutions (see Supplementary Note S1). For all 19 genotypes, the δ^66^Zn values of the plant organs decreased in the order root > stem > leaf. In detail, δ^66^Zn data obtained for the organs ranged between − 1.11‰ and − 0.43‰ for leaves, between − 0.67‰ and + 0.25‰ for stems and between − 0.28‰ and + 0.68‰ for roots (Table 1). The δ^66^Zn values for the total plants ranged between − 0.22‰ and − 0.70‰ (Table 2). There were no strong correlations (R^2^ > 0.5, p < 0.05) between δ^66^Zn values and the Zn concentrations of leaves, stems, roots and total plants.

### Zinc data for plants treated with 0, 5 and 20 µmol L^−1^ CdCl_2_

The total dry masses of the NA 702 seedlings treated with different concentrations of CdCl_2_ (0, 5 and 20 µmol L^−1^) were similar at 1.83 g, 1.70 g, and 1.72 g, respectively (Supplementary Table S7 and Supplementary Fig. S3a,b). In comparison, the 18 remaining genotypes that were treated only with 20 µmol L^−1^ CdCl_2_ produced seedlings with total dry masses that varied between 0.40 g and 2.16 g. These 18 genotypes hence encompass plants that were very similar to the NA 702 seedlings in leaf, stem, root and total plant biomass and others which produced more or less biomass.

**Figure 2:**
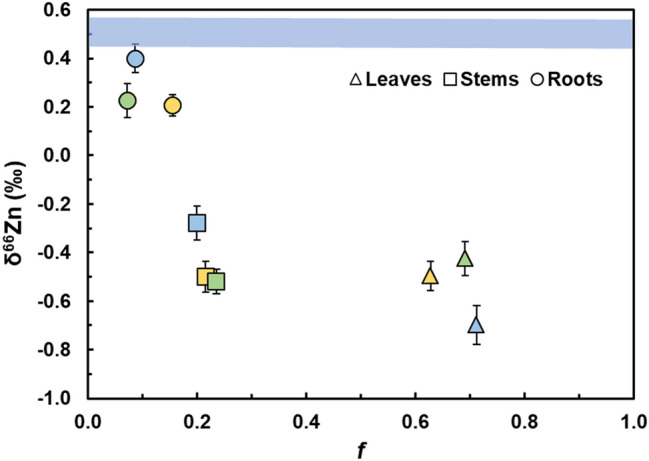
Mean Zn stable isotope compositions (δ^66^Zn) versus mean mass fractions (*f*) of Zn in leaves, stems and roots relative to the total plant Zn inventory for NA 702 plants treated with 0 µmol L^−1^ (yellow, *n* = 3), 5 µmol L^−1^ (green, *n* = 2), and 20 µmol L^−1^ (blue, *n* = 1) CdCl_2_. The error bars denote the 2SD precision determined for multiple analyses of the Zn isotope standard that bracketed the sample runs. The blue line denotes the δ^66^Zn value of + 0.51 ± 0.06‰ for the ZnSO_4_ that was added to the hydroponic solutions.

For the NA 702 seedlings treated with 0, 5 and 20 µmol L^−1^ Cd, the Zn distribution in the organs followed the same pattern seen in the seedlings of different genotypes treated with 20 µmol L^−1^ Cd, with higher Zn concentrations in the stems and leaves compared to the roots (Fig. 2 and Supplementary Table S7, Fig. S4). The NA 702 plants cultured with different Cd treatments (0, 5, and 20 µmol L^−1^) showed no substantial differences in biomass and the distribution and concentration of Zn in the plant organs. The Zn concentration data for the leaves, stems, and roots thereby showed CV values of 13%, 27%, and 33%, respectively. The mean Zn mass fractions present in the organs relative to the total plants were *f*_leaf_ = 66%, *f*_stem_ = 22%, and *f*_root_ = 12% (Supplementary Table S7). Considering the Zn stable isotope data, the δ^66^Zn values of the organs decreased in the same order that was identified for the seedlings of all other genotypes, with root > stem > leaf. The δ^66^ Zn values determined for the cacao organs varied between − 0.70‰ and − 0.43‰ for leaves, between − 0.52‰ and − 0.28‰ for stems, and between + 0.21‰ and + 0.40‰ for roots (Fig. 2, Table 2 and Supplementary Table S7, Fig. S4).

## Discussion

### Stable isotope fractionation during Zn uptake

In this study, the hydroponic plant systems were assumed to be unidirectional, with no efflux of Zn from the plant back to the hydroponic solution. The stable isotope fractionation that occurs during root uptake of Zn can be determined using the following Rayleigh mass-balance equation:

3 $${\Delta }^{66}{\mathrm{Zn}}_{\mathrm{final }-\mathrm{initial }} = {\upvarepsilon }^{66}{\mathrm{Zn}}_{\mathrm{uptake}}*In({f}_{\mathrm{remaining}})$$

where Δ^66^Zn_final-__initial_ represents the isotope difference between the initial and final Zn in the hydroponic solution, ε^66^Zn_uptake_ denotes the stable isotope fractionation factor associated with the uptake of Zn by the cacao plants, and *f*_remaining_ represents the mass fraction of Zn from the hydroponic solution not taken up by the cacao plants. The mean ε^66^Zn_uptake_ for all 19 genotypes was –1.15 ± 0.36‰ (2SD; Table 2), indicating that the cacao plants preferentially took up isotopically light Zn. These similarities in ε^66^Zn_uptake_ across 19 cacao genotypes indicate that the plants of the different genotypes likely employ similar physiological mechanisms to take up Zn from the hydroponic solutions.

Previous studies also noted preferential uptake of light Zn isotopes in both plant-hydroponic^10–12, 57^ and plant-soil systems^9, 13, 16, 18, 19, 24, 57^. In detail, Δ^66^Zn values for fruit and cereal crops, as well as hyperaccumulator plant species, were between − 0.60 and − 0.01‰^9–13, 16, 18, 19, 24, 57^. This preferential uptake of isotopically light Zn has been ascribed to low-affinity transport of free Zn^2+^ ions under Zn sufficient or excess conditions, via ion channels and electrogenic pumps or a combination of both high- and low-affinity transport of Zn^9, 11, 18^. There are exceptions to this observed pattern, where Δ^66^Zn values were positive (as high as + 0.63‰) for *Thlaspi arvense*, *Alyssum murale, Agrostis capillaris* L*., Noccaea caerulescens*, *Arabidopsis helleri*, and wheat^9, 11, 12, 16, 18^. In these cases, Zn uptake may be mediated by high-affinity transport, under Zn deficient conditions, via membrane transporter proteins such as zinc-regulated, iron-regulated transporter-like proteins (ZIPs)^9, 11^. However, the specific conditions at which plants switch between high- and low-affinity transport of Zn have yet to be fully characterised and understood^5, 9^.

The cacao plants of the current study were grown under Zn sufficient conditions. The low-affinity transport hypothesis may therefore explain the negative ε^66^Zn_uptake_ values of the cacao seedlings. In comparison to all other investigated plants, however, cacao plants show the largest Zn stable isotope fractionation during Zn uptake, regardless of genotype (Supplementary Table S8). This suggests that cacao plants may have a different or more pronounced dominant physiological mechanism for Zn uptake compared to other plant species, which is not fully encapsulated by the low-affinity transport hypothesis. A possible reason may be the presence of membrane transporter proteins such as NRAMPs that mediate the uptake of free Zn^2+^ ions into cacao roots, a similar uptake mechanism as hypothesised for free Cd^2+^ ions^58^. It is also not yet clear whether the uptake mechanisms would change under Zn deficient or excess conditions, and this could be investigated in future hydroponic plant studies.

### Stable isotope fractionation during Zn translocation

**Figure 3:**
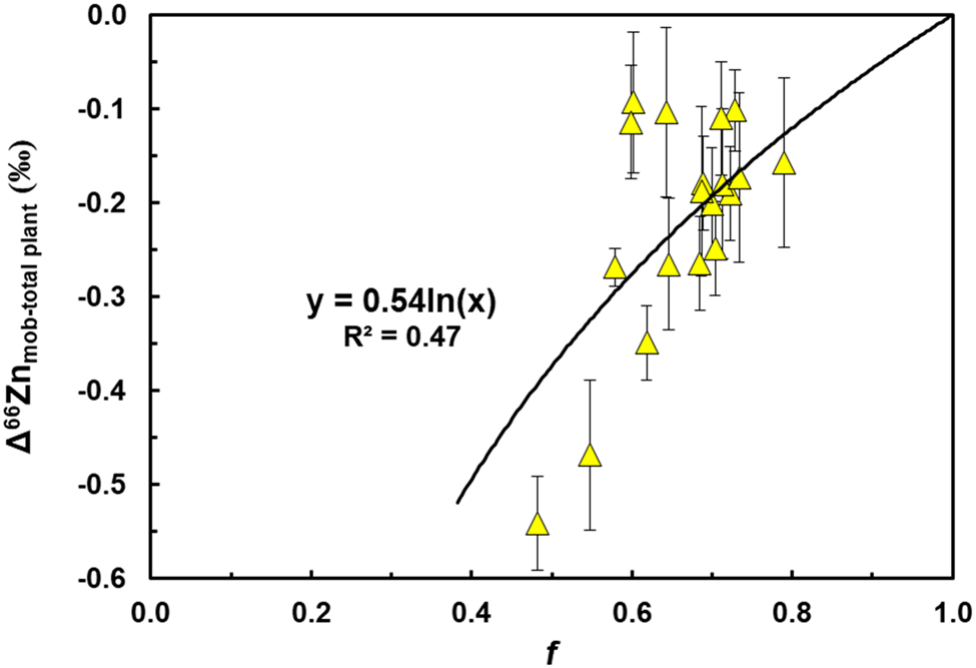
Zinc stable isotope difference (Δ^66^Zn_mob-total plant_) between mobilised Zn in cacao leaves (mob) and total plant Zn inventory versus mass fraction (*f*) of Zn present in cacao leaves for the 19 cacao genotypes treated with 20 µmol L^−1^ CdCl_2_. The solid line is a logarithmic best-fit Rayleigh fractionation trend for the sample data, which is forced through Δ^66^Zn_mob-total plant_ at *f* = 1. This yields an isotope fractionation factor of ε^66^Zn_seq-mob_ =  + 0.54‰ between sequestered Zn and Zn mobilised to leaves.

Partial sequestration (seq) of Zn in roots and stems during unidirectional Zn mobilisation (mob) to the leaves, can produce a stable isotope fractionation that follows a Rayleigh mass balance equation:

4 $${\delta }^{66}{\mathrm{Zn}}_{\mathrm{mob}} = { \delta }^{66}{\mathrm{Zn}}_{\mathrm{total}}+ {\upvarepsilon }^{66}{\mathrm{Zn}}_{\mathrm{seq}-\mathrm{mob}}*\mathrm{ In}({f}_{mob})$$

where $\delta$^66^Zn_mob_ and $\delta$^66^Zn_total_ denote the Zn stable isotope compositions of mobilized Zn in the leaves, and the total plant, respectively, ε^66^Zn_seq-mob_ represents the stable isotope fractionation factor that characterizes the retention and translocation of Zn, and *f*_mob_ represents the mass fraction of mobilised Zn in the leaves relative to the total Zn inventory of the plant. The stable isotope fractionation factor ε^66^Zn_seq-mob_ of the cacao plants is a result of processes that store isotopically heavy Zn in the plant roots and stems and the mobilisation of isotopically light Zn to the leaves. A logarithmic best fit curve for the data in a plot of Δ^66^Zn_mob-total plant_ versus *f*_leaf_, when forced through *f*_leaf_ = 1 at Δ^66^Zn_mob-total plant_ = 0, yields a mean ε^66^Zn_seq-mob_ of + 0.54‰ (Fig. 3). When arithmetically derived, the mean ε^66^Zn_seq-mob_ value is + 0.52 ± 0.36‰ (2SD; Table 2). A single well-defined stable isotope fractionation factor is hence able to fully capture all 19 cacao genotypes, indicating that there are likely only minor differences in the mechanisms that are utilized by the different genotypes to mobilise and retain Zn. The mean ε^66^Zn_seq-mob_ from this study is consistent with previous studies examining Zn translocation in both metal hyperaccumulators and non-hyperaccumulators, in which isotopically light Zn is transferred to aerial parts of the plant^9, 11, 12^. In examining root-to-shoot stable isotope fractionation of Zn, *Thlaspi arvense*, *Noccaea caerulescens* and *Alyssum murale*^11^, *Noccaea caerulescens* and *Silene vulgaris*^9^, and *Arabidopsis halleri* and *Arabidopsis petraea*^12^, had ε^66^Zn_seq-mob_ values of + 0.32‰, + 0.39‰ and + 0.37‰, respectively. This suggests that cacao plants have a mean ε^66^Zn_seq-mob_ value that is identical, within 2SD, to the previous results, in accord with similar partitioning of isotopically light Zn to the aerial parts of the plant. Given the similarity in ε^66^Zn_seq-mob_ values, it is likely that cacao and other plant species employ similar mechanisms to regulate sequestration and translocation of Zn within the plants.

The ε^66^Zn_seq-mob_ values determined in this study indicate that root-to-shoot translocation of Zn in cacao plants is mediated by mechanisms that favour isotopically light Zn, as was previously discussed for other plant species^5, 9, 17, 18, 24^. In cacao plants, as with other plant species, it is likely that once external Zn^2+^ ions have entered the root, the isotopically heavy Zn^2+^ ions in the cytosol preferentially bind with ligands that contain nitrogen- and oxygen-donors^27, 59^, such as nicotianamine and histidine, to form Zn-ligand complexes^60, 61^. This complexed Zn either remains in the cytosol of the cacao roots or is further transferred by membrane transporter proteins, such as metal tolerance proteins (MTPs) on the tonoplast, to vacuoles for storage^5, 9, 17, 18, 24^. As examined in previous plant studies, this likely leads to the enrichment of isotopically light Zn^2+^ ions in the cytosol of cacao roots, which can subsequently be loaded into the xylem by membrane transporter proteins, such as HMAs, for translocation to the cacao stems and leaves^9, 17^.

### Comparison of Zn and Cd stable isotope fractionation in cacao plants

Regardless of the CdCl_2_ concentration added to the hydroponic solutions, the cacao plants preferentially removed isotopically light Zn from the nutrient media. For the NA 702 genotype treated with 0, 5 and 20 µmol L^−1^ CdCl_2_, the mean ε^66^Zn_uptake_ was − 1.06 ± 0.20‰ (2SD; Table 2). The small observed differences in ε^66^Zn_uptake_ values for the different treatments and the similarity of the mean value to that obtained for the 19 genotypes treated with 20 µmol L^−1^ CdCl_2_, (− 1.15 ± 0.36‰, 2SD; Table 2) suggests that the Zn uptake mechanism is not substantially impacted when Cd availability changes. In contrast, the NA 702 plants treated with 0 and 5 µmol L^−1^ CdCl_2_ yielded significantly lower ε^66^Zn_seq-mob_ values than the 19 cacao genotypes that were treated with 20 µmol L^−1^ CdCl_2_, including NA 702 (p < 0.05, Table 2). In detail, the ε^66^Zn_seq-mob_ values for the two low-Cd treatments were + 0.25‰ and + 0.09‰, compared to + 0.53‰ for NA 702 and + 0.52 ± 0.36‰ (2SD) for the other 18 genotypes treated with 20 µmol L^−1^ CdCl_2_. However, irrespective of the CdCl_2_ concentration of the hydroponic solutions, the cacao leaves retained isotopically light Zn compared to the roots.

The ε^66^Zn_seq-mob_ values for the different Cd treatments demonstrate that while cacao plants can maintain Zn homeostasis under different Cd stress conditions, there appear to be differences in the mechanisms that the plants employ to mediate the translocation of Zn when they are exposed to different levels of Cd stress. One possible explanation for this observation is the presence of metallochaperones that maintain Zn homeostasis. Metallochaperones are metal-binding-proteins, which likely preferentially bind with cytosolic Zn^2+^, Cu^2+^, Ni^2+^^62^ and Cd^2+^ ions^63, 64^, and that coordinate with membrane transporter proteins to transfer these heavy metals to different intracellular compartments and plant tissues^65–67^. It is plausible that in cacao plants Zn and Cd share an individual or a series of metallochaperones that are activated together and participate in a *linked* response to heavy metal stress as part of an internal tolerance strategy. The findings of this study may suggest that when excess Cd is introduced and taken up by the plant, in order to maintain Zn homeostasis to ensure plant growth under Cd stress, cacao plants upregulate and activate metallochaperones to chelate free Zn^2+^ ions in the roots and transfer these to aerial plant organs via membrane transporter proteins^65, 67^. In the absence of or under limited Cd stress, these metallochaperones may be downregulated or deactivated. This explanation is in accord with the findings on the formation and sequestration of isotopically heavy ligand-Zn complexes in the cytosol and vacuoles, as metallochaperones could preferentially bind with free cytosolic Zn^2+^ ions that are likely isotopically light and are then transferred to aerial plant organs. However, given the small number of cacao plants grown under low Cd conditions, further work is required to confirm if the inferred differences in ε^66^Zn_seq-mob_ values for cacao plants cultured with different Cd concentrations in the hydroponic solutions are indeed significant. Biological studies are also required to confirm the presence and functionality of metallochaperones that regulate Zn and Cd homeostasis in cacao plants, and the genes that encode them.

When the Zn stable isotope compositions of this study are compared to previously published Cd stable isotope data for the same 19 hydroponically-grown cacao genotypes^46^, inverse isotope fractionation dynamics are observed for the translocation of Zn and Cd (Supplementary Fig. S5). During uptake, however, the 19 cacao genotypes preferentially removed *both* isotopically light Zn and Cd from the hydroponic solutions, indicating there may be similar physiological mechanisms for the uptake of both metals. Moore et al.^46^ determined through stable isotope analyses that the light Cd isotope compositions of the plants relative to the hydroponic solutions may reflect, at least in part, Cd uptake via NRAMP5 membrane transporters. This inference was based on the finding that transgenic yeast encoded with the cacao *TcNRAMP5* gene took up more and isotopically lighter Cd than yeast controls without this gene^46^. Furthermore, Ullah et al.^58^ found in examining the role of NRAMPs in the uptake of Cd and nutritionally vital divalent cations (Fe^2+^, Mn^2+^, Zn^2+^) by cacao, that transgenic yeast strains modified with *TcNRAMP5* genes showed substantial Zn^2+^ transport activity. Hence, it is plausible that cacao plants also utilise similar NRAMP transporters, and in particular NRAMP5, to preferentially take up isotopically light Zn. However, further studies are required to confirm the importance of NRAMP transporters for Zn and Cd uptake by cacao plants.

Once Zn and Cd make their way into the cacao plants, an inverse relationship in stable isotope fractionation was observed, whereby isotopically light Zn and heavy Cd are mobilized to the leaves. This is consistent with the observed stable isotope fractionation for translocation of Zn and Cd in durum wheat and is likely a result of differences in ligand environments^18^. In the cytosol of cacao roots, the isotopically heavy Zn^2+^ ions likely bind with ligands containing oxygen- and nitrogen-donor groups^27, 59^, whereas the isotopically light Cd^2+^ ions likely bind with ligands containing sulphur-donor groups^18, 68^. Given that the heavy and light isotopes of both Zn and Cd preferentially bind to the oxygen- and nitrogen- versus the sulphur-donor groups of ligands, respectively^27, 70^, the observed difference is likely due to the two metals binding to distinct enzymes and/or proteins that contain ligands with the respective electron donors. For instance, isotopically heavy Zn^2+^ ions may bind with ligands attached to organic molecules such as nicotianamine and histidine^27, 59–61^. In contrast, the isotopically light Cd^2+^ ions may bind with the ligands of organic molecules such as glutathione or phytochelatins^18, 68^. The metal–ligand complexes that are formed in such reactions are subsequently immobilized and stored in the cacao roots. The remaining unbound pool of isotopically light Zn^2+^ ions and isotopically heavy Cd^2+^ ions in the cytosol of the root cells are then preferentially loaded into the xylem and transferred to the cacao stems and leaves. The root-to-shoot translocation of isotopically light Zn^2+^ ions and isotopically heavy Cd^2+^ ions could be mediated by similar membrane proteins such as HMAs^9, 17, 46, 70^. However, it is also conceivable that the two metals utilise distinct membrane transporters for root-to-shoot translocation in cacao plants. During root-to-shoot translocation of Zn, in order to maintain homeostasis, it is also likely that under high-Cd stress additional regulation such as the activation of metallochaperones is necessary. This may explain the stronger preference for the transfer of isotopically light Zn to aerial plant organs under high Cd conditions. Further studies are required to better understand the dynamics of the interaction of Zn and Cd in cacao plants, in particular concerning the mechanisms involved in their root-to-shoot translocation.

## Conclusion

All 19 cacao genotypes analysed in this study preferentially removed isotopically light Zn from the hydroponic solutions, with a mean fractionation factor of ε^66^Zn_uptake_ =  − 1.15 ± 0.36‰ (2SD). The uptake of Zn by the cacao plants was not noticeably impacted by exposure to different Cd concentrations (0, 5 and 20 µmol L^−1^ CdCl_2_) in the hydroponic solutions during the growing period. The exposure to different Cd concentrations, however, influenced the translocation of Zn, whereby the stable isotope fractionation was enhanced when the plants were exposed to higher Cd concentrations. In detail, the Zn isotope fractionation factor for translocation from roots to shoots, ε^66^Zn_seq-mob_, was + 0.17 ± 0.13‰ (2SD) for plants grown with 0 and 5 µmol L^−1^ CdCl_2_ in the hydroponic solution, but + 0.52 ± 0.36‰ (2SD) for cacao grown in hydroponic solutions with 20 µmol L^−1^ CdCl_2_. These findings indicate that cacao plants most likely use similar physiological mechanisms for the uptake of both metals but distinct mechanisms for translocation. These interpretations are supported by published Cd stable isotope data for the same cacao plants that were analysed in the current study for Zn^46^. In detail, there is a concurrent enrichment of isotopically light Zn and Cd in the cacao plants in comparison to the hydroponic solutions. In contrast, cacao leaves revealed an inverse isotope fractionation pattern for the two metals, with light Zn isotopes and heavy Cd isotopes enriched in leaves relative to the roots. These systematics may reflect that Zn and Cd share the same membrane transporter proteins for uptake, but that the sequestration and translocation of the two metals is regulated by distinct organic molecules in the cacao roots. Furthermore, the root-to-shoot translocation of Zn may be impacted by metallochaperones when elevated Cd concentrations are present in the plants.

Based on the findings of this study, there are broader implications for Cd stable isotope investigations of cacao plants. An important next step would be to examine the Cd stable isotope compositions of cacao plants cultured at different Zn conditions. Of particular interest is whether higher Zn concentrations in the nutrient media alter the uptake and translocation of Cd, both in hydroponics– and soil–plant systems, including for mature cacao plants. Such experiments may help to elucidate whether similar or distinct mechanisms are responsible for Zn and Cd homeostasis in cacao plants. In particular, if such work confirms that Zn and Cd share similar uptake mechanisms, this may enable the use of Zn amendments to soils to reduce the overall Cd uptake of cacao by a competition mechanism. Furthermore, the contrasting stable isotope systematics of the two metals within the plants suggest it may be possible to mitigate the high Cd concentrations of cacao beans without affecting essential Zn, via knock-out or overexpression of genes responsible for encoding proteins that assist in the translocation of Cd to above-ground parts or in the sequestration in roots, respectively. Also desirable are X-ray absorption spectroscopy studies to further constrain Zn and Cd speciation and ligand-binding environments in different cacao plant organs and investigations of genes that are likely to play a role in the uptake, sequestration and mobilisation of the two metals.

## Supplementary Information

Supplementary Information.

## Data Availability

All generated or analysed data during this study are included in this published article (and its Supplementary Information files).
