# Publisher Correction: An investigation of zinc isotope fractionation in cacao (*Theobroma cacao* L.) and comparison of zinc and cadmium isotope compositions in hydroponic plant systems under high cadmium stress

**DOI:** 10.1038/s41598-023-34474-4

**Published:** 2023-05-09

**Authors:** Elnaz Barati, Rebekah E. T. Moore, Ihsan Ullah, Katharina Kreissig, Barry J. Coles, Jim M. Dunwell, Mark Rehkämper

**Affiliations:** 1 Department of Earth Science and Engineering, Imperial College London, London, UK; 2 School of Agriculture, Policy and Development, University of Reading, Reading, UK

Correction to: *Scientific Reports* 10.1038/s41598-023-30899-z, published online 22 March 2023

The original version of this Article contained an error in the Methods section, under the subheading ‘Ethical approval’,

“All research complied with all relevant institutional, national, and international guidelines and legislation. Please note we have moved the section “Ethical approval” to the end of the methods, as per house style. We acknowledge and agree with changes in format for "Ethical Approval".”

now reads:

“All research complied with all relevant institutional, national, and international guidelines and legislation.”

The original Article has been corrected.

