# Supplementary material for: An investigation of zinc isotope fractionation in cacao (Theobroma cacao L.) and comparison of zinc and cadmium isotope compositions in hydroponic plant systems under high cadmium stress
Source: Sci Rep. 2023 Mar 22;13:4682. doi: 10.1038/s41598-023-30899-z (PMC10033898; doi:10.1038/s41598-023-30899-z)
Supplement: Supplementary file 1 — Supplementary Information. [file 41598_2023_30899_MOESM1_ESM.pdf]

Supplementary Information

An investigation of zinc isotope fractionation in cacao (*Theobroma cacao* L.) and comparison of zinc and cadmium isotope compositions in hydroponic plant systems under high cadmium stress

***Elnaz Barati,** eb1818@ic.ac.uk**,** Department of Earth Science and Engineering, Imperial College London, London SW7 2BP, UK,

Rebekah E. T. Moore, r.moore13@imperial.ac.uk, Department of Earth Science and Engineering, Imperial College London, London SW7 2BP, UK,

Ihsan Ullah, i.ullah@reading.ac.uk, School of Agriculture, Policy and Development, University of Reading, Reading RG6 6EU, UK,

Katharina Kreissig, k.kreissig@imperial.ac.uk, Department of Earth Science and Engineering, Imperial College London, London SW7 2BP, UK,

Barry J. Coles, b.coles@imperial.ac.uk, Department of Earth Science and Engineering, Imperial College London, London SW7 2BP, UK,

Jim M. Dunwell, j.m.dunwell@reading.ac.uk, School of Agriculture, Policy and Development, University of Reading, Reading RG6 6EU, UK,

Mark Rehkämper, markrehk@imperial.ac.uk, Department of Earth Science and Engineering, Imperial College London, London SW7 2BP, UK

**Corresponding author*

Table of Contents

Table S1. Cacao clone names, accession numbers and donor organizations for the cacao germplasms used in this study. **3**

Table S2. Composition of the half-strength Hoagland solutions with additional $CdCl_2$. **4**

Table S3. Zinc concentrations ([Zn]; mg kg^{-1}) and stable isotope compositions ($\delta^{66}Zn$) for quality control materials. **5**

Table S4. Summary of mass fractions (*f*), Zn concentrations ([Zn]; mg kg^{-1}) and Zn stable isotope compositions (δ^{66} Zn) for leaves, stems and roots determined for biological and analytical replicates. **6**

Table S5. Dry weights (g) of the different plant organs treated with 20 µmol L^{-1} $CdCl_2$. **7**

Table S6. Summary of Zn concentration data (mg kg^{-1}) for cacao leaves from this study and the literature. **8**

Table S7. Summary of mean dry weights (g), mass fractions (*f*), Zn concentrations ([Zn]; mg kg^{-1}) and Zn stable isotope compositions (δ^{66} Zn) of leaves, stems and roots and total plant dry weights (g) for seedlings of the NA 702 genotype treated with 0, 5, and 20 µmol L^{-1} $CdCl_2$. **9**

Table S8. Summary of $\Delta^{66}Zn$ for uptake, and Zn stable isotope compositions (δ^{66} Zn) for root, shoot and leaf published for different plant species. **10**

Note S1: Depletion of Zn in the hydroponic solutions. **11**

Figure S1. Zinc stable isotope compositions ($\delta^{66}Zn$) versus Zn concentrations ([Zn]; mg kg^{-1}) for leaves, stems, roots, and whole plants of the 19 cacao genotypes treated with 20 µmol L^{-1} $CdCl_2$. **12**

Figure S2. Leaf Zn concentrations ([Zn]; mg kg^{-1}) versus total plant Zn concentrations (mg kg^{-1}) for the 19 cacao genotypes. **13**

Figure S3. (a) Dry weights (g) and (b) mass fraction (%) for organs of NA 702 plants cultured with 0, 5 and 20 µmol L^{-1} $CdCl_2$ and mean results for the other 18 cacao genotypes cultured with 20 µmol L^{-1} $CdCl_2$. **14**

Figure S4. Mean Zn stable isotope compositions ($\delta^{66}Zn$) for leaves, stems and roots versus mean Zn concentrations ([Zn]; mg kg^{-1}) of the NA702 seedlings treated with 0 µmol L^{-1} (yellow), 5 µmol L^{-1} (green) and 20 µmol L^{-1} (blue) $CdCl_2$. **15**

Figure S5. Cadmium stable isotope compositions ($\delta^{114}Cd$) versus Zn stable isotope composition ($\delta^{66}Zn$) for roots and leaves of the 19 cacao genotypes treated with 20 µmol L^{-1} $CdCl_2$. **16**

References **17-18**

Table S1. Cacao clone names, accession numbers and donor organizations of the cacao germplasms used in this study.

Cacao Clones (Genotype) Name	Accession number (ICQC, R)	Donor Collection
**B 5/7 [POU]**	RUQ 522	ICG, T
**Catie 1000**	RUQ	CIRAD
**CC 41**	RUQ	ICG, T
**CL 19/10**	RUQ	ICG, T
**GU 207/H**	RUQ	CIRAD
**GU 236/V**	RUQ	CIRAD
**IMC 27**	RUQ	ICG, T
**LP 1/41 [POU]**	RUQ	ICG, T
**Matina 1-7**	RUQ	ICG, T
**NA 702**	RUQ	ICG, T
**PNG 340**	RUQ	CIRAD
**Pound 12/A [POU]**	RUQ	ICG, T
**RB 46**	RUQ	CATIE
**RIM 179**	RUQ	CATIE
**SCA 9**	RUQ	ICG, T
**SPA 9 [COL]**	RUQ	ICG, T
**TARS 31**	RUQ	USDA-TARS
**TSA 654**	RUQ	CEPLAC/CEPEC
**U 70 [PER]**	RUQ	UNAS

CATIE	Centro Agronómico Tropical de Investigión y Enseñanza (Costa Rica)
CEPLAC	Comissão Executiva do Plano da Lavoura Cacaueira (Brazil)
CIRAD	Centre de cooperation international en recherché agronomique pour le développement (France)
ICG, T	International Cocoa Genebank (Trinidad)
ICQC, R	International Cocoa Quarantine Centre, Reading (UK)
UNAS	Universidad Nacional Agraria de la Selva (Peru)
USDA-TARS	United States Department of Agriculture, Tropical Crops and Germplasm Research, Mayaguez (Puerto Rico)

Table S2. Composition of the half-strength Hoagland solution including additional $CdCl_2$.

Component	Species	Concentration	
		mg L^{-1}	µmol L^{-1}
**Major nutrients**			
**KH_2PO_4**	K^+	19.55	500
	PO_4^{2-}	47.49	500
	H^+	3.002	1500
**KNO_3**	K^+	97.75	2500
	NO_3^-	155.0	2500
**$Ca(NO_3)_2 \cdot 4H_2O$**	Ca_2^+	100.2	2500
	NO_3^-	310.0	5000
**$MgSO_4 \cdot 7H_2O$**	Mg^{2+}	24.31	1000
	SO_4^{2-}	96.07	1000
**Micronutrients**			
**H_3BO_3**	H_3BO_3	1.430	23.1
**Fe-EDTA[a]**	Fe^{3+}	2.383	42.7
	$EDTA^{4-}$	12.30	42.7
**$MnCl_2 \cdot 4H_2O$**	Mn^{2+}	0.253	4.6
	Cl^-	0.326	9.2
**$ZnSO_4 \cdot 7H_2O$[a]**	Zn^{2+}	0.025	0.38
	SO_4^{2-}	0.037	0.38
**$CuSO_4 \cdot 5H_2O$**	Cu^{2+}	0.010	0.2
	SO_4^{2-}	0.015	0.2
**$Na_2MoO_4 \cdot 2H_2O$**	Na^+	0.002	0.1
	MoO_4^{2-}	0.003	0.04
**$CdCl_2$[b]**	Cd^{2+}	2.248	20.0
	Cl-	1.418	20.0

[a]$C_{10}H_{12}FeN_2O_8$. [b]After 28 day growing period, $CdCl_2$ was introduced to the hydroponic solutions for an additional 14 days.

Table S3. Zinc concentrations ([Zn]; mg kg^{-1}) and stable isotope compositions ($\delta^{66}Zn$) for quality control materials.

			[Zn] ± SD mg kg^{-1}		δ^{66} Zn ± 2SD ‰	
**Material**	**Type**	**n**	**This study**	**Literature**	**This study**	**Literature**
**London Zinc**	Pure Zn solution	30	-	-	+0.10 ±0.06	+0.12 ± 0.06[a]
**NIST SRM 1570a**	Spinach leaf	19	79 ± 6	82 ± 4[b]	+0.41 ±0.06	-

All stable isotope compositions (δ^{66}) are reported relative to JMC-Lyon Zn. n represents the number of individual samples that were analysed. [a]The $\delta^{66}Zn$ ± 2SD (‰) is the mean value reported in Archer et al. (2017). [b] The Zn concentration data outlined in the original certificate. SD represents standard deviation. 2SD for individual samples represents the analytical precision of the Zn stable isotope data determined from multiple analyses of the Zn isotope standard (AA-ETH-Zn) that bracketed the sample runs.

Table S4. Summary of mass fractions (*f*), Zn concentrations ([Zn]; mg kg^{-1}) and Zn stable isotope compositions (δ^{66}Zn) for leaves, stems and roots determined for biological and analytical replicates.

Cacao Clones (Genotype)	Replicate	Leaf				Stem				Root				Total Plant
		f	[Zn] mg kg^{-1}	δ^{66}Zn ‰	2SD ‰	*f*	[Zn] mg kg^{-1}	δ^{66}Zn ‰	2SD ‰	*f*	[Zn] mg kg^{-1}	δ^{66}Zn ‰	2SD ‰	[Zn] mg kg^{-1}
**CC 41[a]**	1	0.66	24	−0.82	0.06	0.25	18	−0.19	0.06	0.10	16	0.58	0.06	21
	2	0.73	22	−0.75	0.02	0.18	19	−0.14	0.08	0.09	14	0.44	0.08	20
	3	0.77	35	−0.41	0.07	0.17	19	−0.20	0.08	0.06	11	0.50	0.08	27
**Mean**	-	**0.72**	**27**	**−0.66**	-	**0.20**	**19**	**-0.18**	-	**0.08**	**14**	**0.51**	-	**23**
**1SD**	-	**0.05**	**7**	-	-	**0.04**	**1**	-	-	**0.02**	**2**	-	-	**4**
**2SD**	-	-	-	**0.36**	-	-	-	**0.05**	-	-	-	**0.11**	-	-
**GU 207/H[a]**	1	0.68	27	−0.43	0.06	0.15	27	−0.44	0.06	0.17	18	0.33	0.03	25
	2	0.77	36	−0.43	0.03	0.16	32	−0.39	0.08	0.06	13	0.48	0.03	32
	3	0.73	33	−0.44	0.04	0.14	26	−0.42	0.07	0.12	18	0.38	0.08	29
**Mean**	-	**0.73**	**32**	**−0.43**	-	**0.15**	**28**	**−0.42**	-	**0.12**	**16**	**0.40**	-	**29**
**1SD**	-	**0.04**	**4**	-	-	**0.01**	**3**	-	-	**0.04**	**2**	-	-	**3**
**2SD**	-	-	-	**0.01**	-	-	-	**0.05**	-	-	-	**0.15**	-	-
**Matina 1-7[b]**	1	0.65	20	−0.68	0.09	0.27	23	−0.63	0.08	0.08	13	0.01	0.08	20
	1	0.55	19	−0.78	0.06	0.35	33	−0.63	0.04	0.09	16	−0.04	0.08	22
**Mean**	-	**0.60**	**20**	**−0.73**	-	**0.31**	**28**	**−0.63**	-	**0.09**	**15**	**−0.02**	-	**21**
**1SD**	-	**0.05**	**1**	-	-	**0.04**	**5**	-	-	**0.01**	**2**	-	-	**1**
**2SD**	-	-	-	**0.14**	-	-	-	**0**	-	-	-	**0.07**	-	-

[a] Biological replicates; single digests of leaf, stem and root samples from three separate plants of CC 41 and GU 207/H. [b] Analytical replicates; duplicate digests of leaf, stem and root samples from a single plant of Matina 1-7. SD represents the standard deviation; 1SD is used for *f* and Zn concentrations ([Zn]) and 2SD for Zn stable isotope compositions (δ^{66}Zn). 2SD for individual samples represents the analytical precision of the Zn stable isotope data determined from multiple analyses of the Zn isotope standard that bracketed the sample runs.

Table S5. Dry weights (g) of the organs from plants treated with 20 μmol L^{-1} $CdCl_2$.

Genotype	Leaf g	Stem g	Root g	Total Plant g
**B 5/7 [POU]**	0.554	0.246	0.092	0.892
**Catie 1000**	0.304	0.122	0.127	0.553
**CC 41[a]**	0.584	0.220	0.125	0.929
**CL 19/10**	1.361	0.289	0.159	1.809
**GU 207/H[a]**	0.393	0.094	0.115	0.602
**GU 236/V**	0.332	0.089	0.144	0.565
**IMC 27**	0.301	0.147	0.132	0.580
**LP 1/41 [POU]**	0.635	0.259	0.101	0.995
**Matina 1-7**	1.387	0.499	0.269	2.155
**NA 702**	1.181	0.341	0.198	1.720
**PNG 340**	0.940	0.551	0.206	1.697
**Pound 12/A [POU]**	0.525	0.139	0.121	0.785
**RB 46**	0.512	0.140	0.134	0.786
**RIM 179**	1.298	0.334	0.299	1.931
**SCA 9**	0.668	0.262	0.122	1.052
**SPA 9 [COL]**	0.535	0.218	0.127	0.880
**TARS 31**	0.534	0.181	0.280	0.995
**TSA 654**	0.616	0.153	0.138	0.907
**U 70 [PER]**	0.247	0.084	0.068	0.399
**Mean**	**0.679**	**0.230**	**0.156**	**1.065**
**1SD**	**0.370**	**0.131**	**0.065**	**0.527**

[a]Mean values for the single leaf, stem, and root aliquots of three separate plants of the CC 41 and GU 207/H genotypes (n = 3). SD represents the standard deviation.

Table S6. Summary of Zn concentration data (mg kg^{-1}) for cacao leaves from this study and the literature.

Reference	Country	Cacao leaf Zn concentrations (mg kg^{-1})
This Study	-	14 – 63
Gramlich et al. (2018)	Honduras	17 – 381
Barraza et al. (2021)	Ecuador	17 – 263
Gramlich et al. (2017)	Bolivia	36.8 – 48.8

Table S7. Summary of mean dry weights (g), mass fractions (*f*), Zn concentrations ([Zn]; mg kg^{-1}) and Zn stable isotope compositions (δ^{66} Zn) of leaves, stems and roots and total plant dry weights (g) for seedlings of the NA 702 genotype treated with 0, 5, and 20 µmol L^{-1} $CdCl_2$.

**$CdCl_2$ µmol L^{-1}**	**n**	**Leaf**					**Stem**					**Root**					**Total Plant**
		**Dry weight g**	***f***	**[Zn] mg kg $^{-1}$**	**δ^{66}Zn ‰**	**2SD ‰**	**Dry weight g**	***f***	**[Zn] mg kg $^{-1}$**	**δ^{66}Zn ‰**	**2SD ‰**	**Dry weight g**	***f***	**[Zn] mg kg $^{-1}$**	**δ^{66}Zn ‰**	**2SD ‰**	**Dry weight g**
**0**	3	1.22	0.63	15	−0.50	0.06	0.38	0.22	18	−0.50	0.06	0.23	0.16	20	0.21	0.04	1.83
**5**	2	1.19	0.69	15	−0.43	0.07	0.31	0.24	21	−0.52	0.05	0.20	0.07	10	0.23	0.07	1.70
**20**	1	1.18	0.71	16	−0.70	0.08	0.34	0.20	16	−0.28	0.07	0.20	0.09	12	0.40	0.06	1.72
**Mean**		**1.20**	**0.66**	**16**	**−0.51**	**-**	**0.35**	**0.22**	**18**	**−0.46**	**-**	**0.21**	**0.12**	**15**	**0.25**	**-**	**1.77**
**1SD**		**0.06**	**0.05**	**2**	**-**	**-**	**0.06**	**0.03**	**5**	**-**	**-**	**0.03**	**0.05**	**5**	**-**	**-**	**0.09**
**2SD**		**-**	**-**	**-**	**0.29**	**-**	**-**	**-**	**-**	**0.21**	**-**	**-**	**-**	**-**	**0.14**	**-**	**-**

n represents the number of individual samples that were digested and analysed. SD represents standard deviation; 1SD is used for dry weight, *f* and Zn concentrations ([Zn]) and 2SD for Zn stable isotope compositions (δ^{66}Zn). 2SD for individual samples represents the analytical precision of the Zn stable isotope data determined from multiple analyses of the Zn isotope standard that bracketed the sample runs.

Table S8. Summary of $\Delta^{66}Zn$ for uptake, and Zn stable isotope compositions (δ^{66} Zn) for root, shoot and leaf published for different plant species.

Reference	Plant Species	Zn concentrations in nutrient sources	$\Delta^{66}Zn$ represents	$\Delta^{66}Zn$ ‰[a]	Root $\delta^{66}Zn$ ‰	Shoot $\delta^{66}Zn$ ‰	Leaves $\delta^{66}Zn$ ‰
This Study	*Theobroma cacao* L.	0.38 µmol L^{-1}	Total plant–hydroponic solution	−1.21 to −0.73 and *−1.49 to −0.86*	−0.28 to +0.68	-	−1.11 to −0.43
Aucour et al. (2011)	*Arabidopsis halleri* and *Arabidopsis petraea*	10 and 250 µmol L^{-1}	Total plant–hydroponic solution	−0.36 to +0.00	+0.19 to +0.96	−0.54 to +0.16	-
Aucour et al. (2017)	*Typha latifolia*	2089 mg kg^{-1}[g]	Total plant–soil	−0.50[b]	+0.03[b]	-	−0.32[b]
Aucour et al. (2015)	*Phalaris arundinacea*	2129 mg kg^{-1}[g]	Total plant–soil	−0.60[b]	+0.24[b]	-	−0.19[b]
Deng et al. (2014)	*Thlaspi arvense, Alyssum murale and Noccaea caerulescens*	2 and 50 µmol L^{-1}	Total plant–hydroponic solution	−0.23 to +0.20[b]	+0.17 to +1.12	−0.59 to +0.23	-
Tang et al. (2016)	*Noccaea caerulescens* and *Thlaspi arvense*	0.02, 1, 5 and 50 µmol L^{-1}	Total plant–hydroponic solution	−0.26 to −0.06[b]	+0.14 to +0.82	−0.26 to +0.13	-
Houben et al. (2014)	*Agrostis capillaris* L.	9470 and 56600 mg kg^{-1}[g]	Total plant–soil leachate	−0.04 and +0.02[f]	+0.17 to +0.36[f]	−0.10 to −0.05[f]	-
Tang et al. (2012)	*Noccaea caerulescens and Silene vulgaris*	107, 115, 2220 and 2396 mg kg^{-1}[g]	Total plant–soil	−0.05 to +0.63[b]	+0.68 to +0.99[b]	-	+0.19 to +0.88[b]
Smolders et al. (2013)	*Lycopersicon esculentum* L. (tomato)	<0.03, 0.9 and 1.0 µmol L^{-1}	Total plant–hydroponic solution	−0.33 to −0.06	+0.16 to +0.33[b]	−0.36 to +0.24[b]	-
Arnold et al. (2015)	*Oryza sativa* L. (rice)	26 mg kg^{-1}[h]	Total plant above ground–soil leachate	−0.27 and −0.08[c]	-	+0.61 and +0.73[c]	-
Wiggenhauser et al. (2018)	*Triticum aestivum* L. (wheat)	0.4 and 10.9 mg kg^{-1}[h]	Total plant–soil extract	−0.33 and +0.13[d]	+0.56 and +0.63[d]	-	+0.10 to +0.72[e]

All Zn stable isotope compositions ($\delta^{66}Zn$) are reported relative to JMC-Lyon Zn. [a] non-italicized values represent isotopic differences, whereas italicized values represent the isotope fractionation factor ($\varepsilon^{66}Zn$) for uptake. [b] mean values. [c] mean values for rice grown in anaerobic and aerobic soils, respectively. [d] mean values for wheat grown in soil from two different sites. [e] range of mean values for flag and senescent leaves for wheat. [f] mean values for *Agrostis capillaris* L. grown in soil from two different sites. [g] represents total Zn concentrations in soils. [h] represents total Zn concentration in soil leachates or extracts.

Note S1. Depletion of Zn from the hydroponic solutions.

Plants were grown in 3 L hydroponic systems containing 4 plants of the same genotype (see main text for further experimental details). The total Zn mass taken up by one plant from each system was calculated based on the Zn concentrations in each organ and their dry mass. For two of the 19 genotypes (CC 41 and GU 207/H), the total Zn mass was calculated for three plants and an average was taken. Given the good agreement between the total Zn masses for the replicates of CC 41 and GU 207/H, all plants in each system were assumed to take up the same mass of Zn, and therefore the total Zn mass of one plant was multiplied by four. This total mass of Zn that was removed from the hydroponic solution was then converted to a percentage of the total Zn mass available in the hydroponic systems (equation S1):

$$\text{Hydroponic Zn depletion (\%)} = \left(\frac{\text{Total Zn of 4 plants}}{\text{Total Zn in hydroponic substrate}}\right) \text{x } 100 \qquad \text{(S1)}$$

The percentages (21±8%, 1SD) were then used to calculate the initial and final Zn stable isotope compositions of the hydroponic solutions, and ultimately, the Zn stable isotope fractionation factor ε^{66}Zn during root uptake (see main text for details).

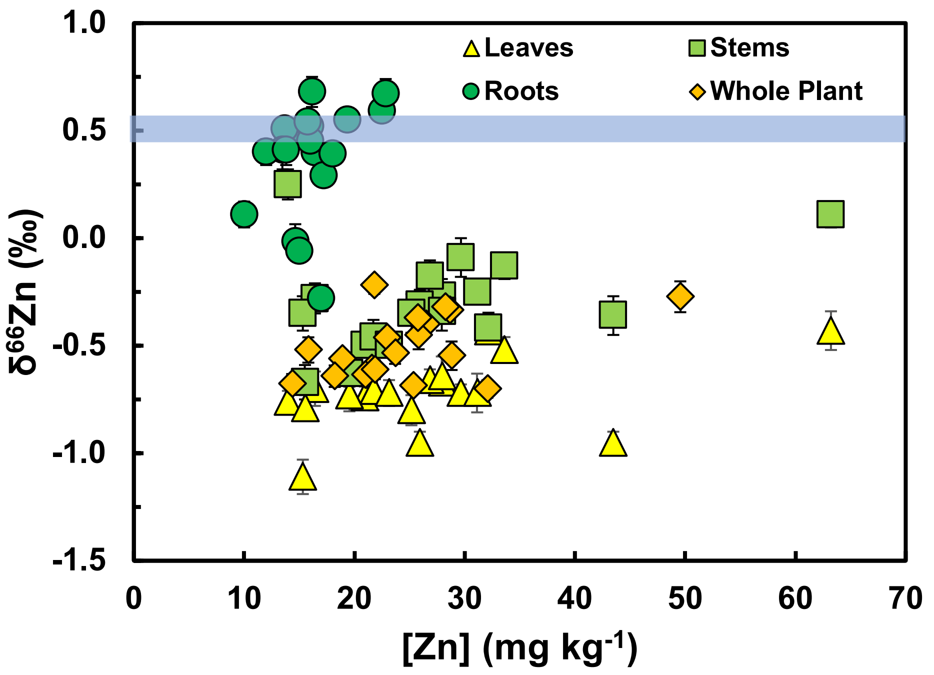

Figure S1. Zinc stable isotope compositions (δ^{66}Zn) versus Zn concentrations ([Zn]; mg kg^{-1}) for leaves, stems, roots, and whole plants of the 19 cacao genotypes treated with 20 µmol L^{-1} $CdCl_2$. The error bars denote the 2SD precision determined for multiple analyses of the Zn isotope standard that bracketed the sample runs. The blue line denotes the δ^{66}Zn value of +0.51±0.06‰ for the $ZnSO_4$ that was added to the hydroponic solutions.

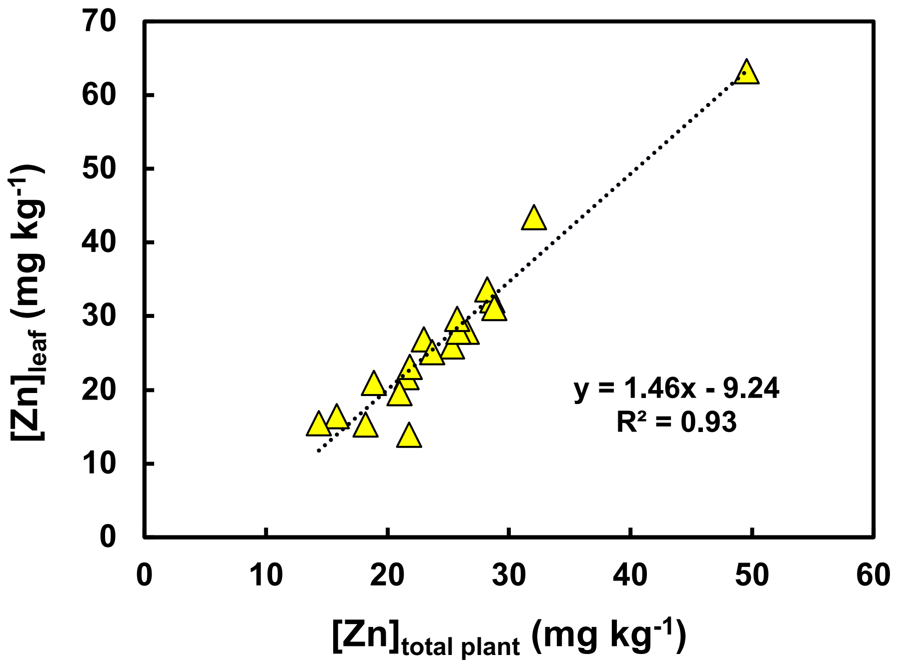

Figure S2. Leaf Zn concentrations ([Zn]; mg kg^{-1}) versus total plant Zn concentrations (mg kg^{-1}) for the 19 cacao genotypes.

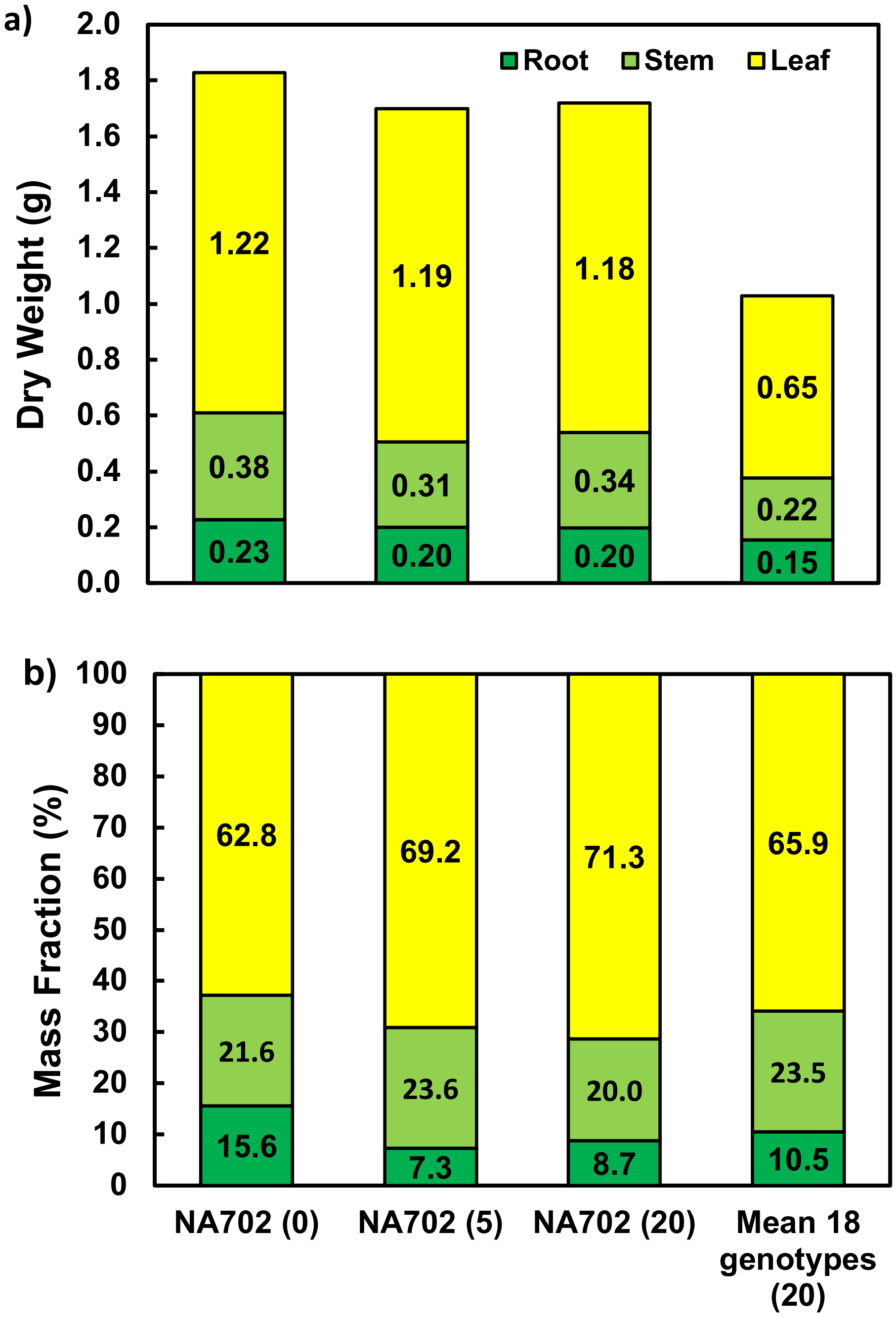

Figure S3. (a) Dry weights (g) and (b) mass fraction (%) for organs of NA 702 plants cultured with 0, 5 and 20 μmol L^{-1} $CdCl_2$ and mean results for the other 18 cacao genotypes cultured with 20 μmol L^{-1} $CdCl_2$. The notations (0), (5), (20) represent NA 702 plants treated with 0, 5, and 20 μmol L^{-1} $CdCl_2$, respectively.

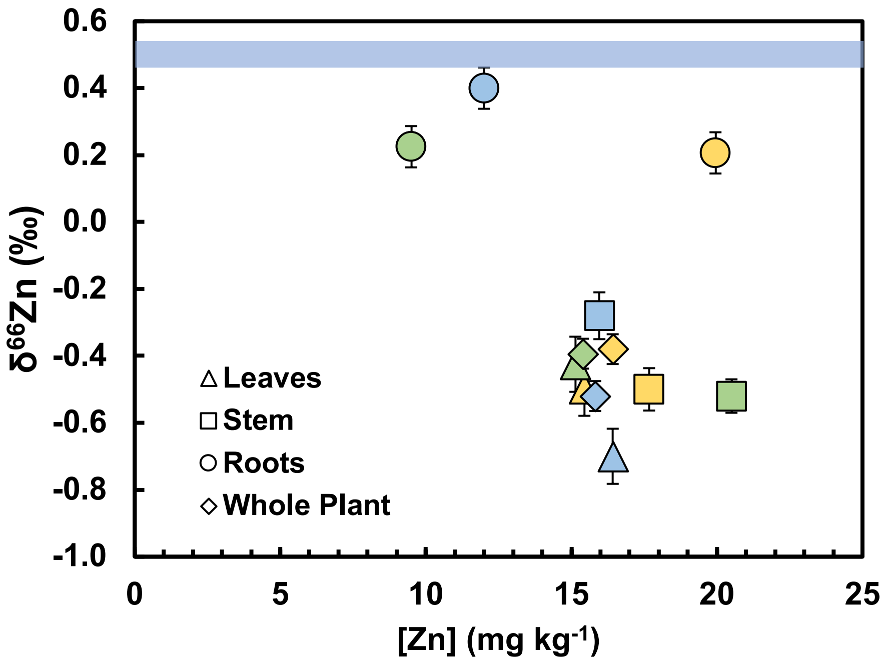

Figure S4. Mean Zn stable isotope compositions ($\delta^{66}Zn$) for leaves, stems and roots versus mean Zn concentrations ([Zn]; mg kg^{-1}) of the NA702 seedlings treated with 0 µmol L^{-1} (yellow), 5 µmol L^{-1} (green) and 20 µmol L^{-1} (blue) $CdCl_2$. The error bars denote the 2SD precision determined for multiple analyses of the Zn isotope standard that bracketed the sample runs. The blue line denotes the $\delta^{66}Zn$ value of +0.51±0.06‰ for the $ZnSO_4$ that was added to the hydroponic solutions.

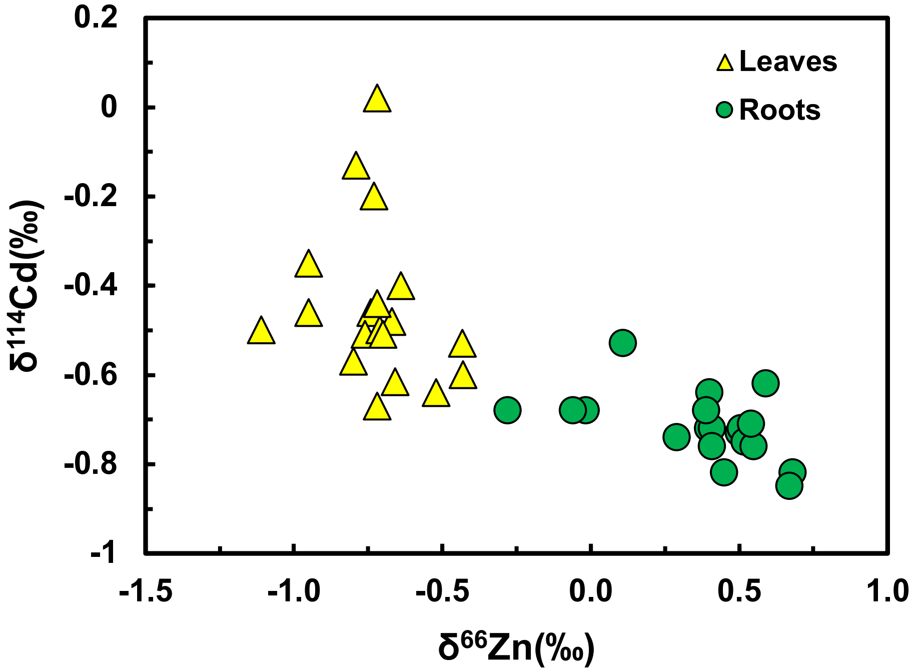

Figure S5. Cadmium stable isotope compositions (δ^{114}Cd) versus Zn stable isotope composition (δ^{66}Zn) for roots and leaves of the 19 cacao genotypes treated with 20 µmol L^{-1} $CdCl_2$. The Cd isotope data is from Moore et al. (2020).

References

Archer, C., Andersen, M. B., Cloquet, C., Conway, T. M., Dong, S., Ellwood, M., Moore, R., Nelson, J., Rehkämper, M., Rouxel, O., Samanta, M., Shin, K.-C., Sohrin, Y., Takano, S., & Wasylenki, L., 2017. Inter-calibration of a proposed new primary reference standard AA-ETH Zn for zinc isotopic analysis. *Journal of Analytical Atomic Spectrometry*, 32(2), pp.415–419.

Arnold, T., Markovic, T., Kirk, G. J. D., Schönbächler, M., Rehkämper, M., Zhao, F. J., & Weiss, D. J., 2015. Iron and zinc isotope fractionation during uptake and translocation in rice (*Oryza sativa*) grown in oxic and anoxic soils. *Comptes Rendus Geoscience*, 347(7-8), pp.397–404.

Aucour, A. M., Pichat, S., Macnair, M. R., & Oger, P., 2011. Fractionation of stable zinc isotopes in the zinc hyperaccumulator *Arabidopsis halleri* and nonaccumulator *Arabidopsis petraea*. *Environmental Science & Technology*, 45(21), pp.9212–9217.

Aucour, A., Bedell, J., Queyron, M., Tholé, R., Lamboux, A., & Sarret, G., 2017. Zn speciation and stable isotope fractionation in a contaminated urban wetland soil–*Typha latifolia* system. *Environmental Science & Technology*, 51(15), pp.8350–8358.

Aucour, A.-M., Bedell, J.-P., Queyron, M., Magnin, V., Testemale, D., & Sarret, G., 2015. Dynamics of zn in an urban wetland soil–plant system: Coupling isotopic and EXAFS approaches. *Geochimica et Cosmochimica Acta*, 160, pp.55–69.

Barraza, F., Schreck, E., Uzu, G., Lévêque, T., Zouiten, C., Boidot, M. & Maurice, L., 2021. Beyond cadmium accumulation: Distribution of other trace elements in soils and cacao beans in Ecuador. *Environmental Research.* 192, pp. 110241.

Deng, T.-H.-B., Cloquet, C., Ye-Tao, T., Sterckeman,T., Echevarria, G., Estrade, N., Jean-Louis Morel, J-L. & Qiu, R-L., 2014. Nickel and zinc isotope fractionation in hyperaccumulating and nonaccumulating plants. *Environmental Science & Technology*, 48(20), pp.11926–11933.

Gramlich, A., Tandy, S., Andres, C., Chincheros Paniagua, J., Armengot, L., Schneider, M. & Schulin, R., 2017. Cadmium uptake by cocoa trees in agroforestry and monoculture systems under conventional and organic management. *The Science of the Total Environment.* 580, pp. 677-686.

Gramlich, A., Tandy, S., Gauggel, C., López, M., Perla, D., Gonzalez, V. & Schulin, R., 2018. Soil cadmium uptake by cocoa in Honduras. *The Science of the Total Environment.* 612, pp. 370-378.

Houben, D., Sonnet, P., Tricot, G., Mattielli, N., Couder, E., & Opfergelt, S., 2014. Impact of root-induced mobilization of zinc on stable Zn isotope variation in the soil–plant system. *Environmental Science & Technology*, 48(14), pp.7866–7873.

Moore, R., Ullah, I., de Oliveira, V.H., Hammond, S.J., Strekopytov, S., Tibbett, M., Dunwell, J.M., & Rehkamper, M., 2020. Cadmium isotope fractionation reveals genetic variation in Cd uptake and translocation by *Theobroma cacao* and role of natural resistance-associated macrophage protein 5 and heavy metal ATPase-family transporters. *Horticulture Research* 7, 71.

Smolders, E., Versieren, L., Shuofei, D., Mattielli, N., Weiss, D., Petrov, I., & Degryse, F., 2013. Isotopic fractionation of Zn in tomato plants suggests the role of root exudates on Zn uptake. *Plant and Soil*, 370(1-2), pp.605–613.

Tang, Y.-T., Cloquet, C., Sterckeman, T., Echevarria, G., Carignan, J., Qiu, R.-L., & Morel, J.-L. 2012. Fractionation of stable zinc isotopes in the field-grown zinc hyperaccumulator *Noccaea caerulescens* and the zinc-tolerant plant *Silene vulgaris*. *Environmental Science & Technology*, 46(18), pp.9972–9979.

Tang, Y.-T., Cloquet, C., Deng, T.-H.-B., Sterckeman, T., Echevarria, G., Yang, W.-J., Morel, J.-L., & Qiu, R.-L., 2016. Zinc isotope fractionation in the hyperaccumulator *Noccaea caerulescens* and the nonaccumulating plant *Thlaspi arvense* at low and high Zn Supply. *Environmental Science & Technology*, 50(15), pp.8020–8027.

Wiggenhauser, M., Bigalke, M., Imseng, M., Keller, A., Archer, C., Wilcke, W., & Frossard, E., 2018. Zinc isotope fractionation during grain filling of wheat and a comparison of zinc and cadmium isotope ratios in identical soil-plant systems. *New Phytologist*, 219(1), pp.195–205.
